# Whole‐genome sequencing reveals the artificial selection and local environmental adaptability of pigeons (*Columba livia*)

**DOI:** 10.1111/eva.13284

**Published:** 2021-08-05

**Authors:** Haobin Hou, Xiaoliang Wang, Weixing Ding, Changfeng Xiao, Xia Cai, Wenwei Lv, Yingying Tu, Weimin Zhao, Junfeng Yao, Changsuo Yang

**Affiliations:** ^1^ Shanghai Academy of Agricultural Sciences Shanghai China; ^2^ National Poultry Engineer Research Center Shanghai China; ^3^ Shanghai Jinhuang Pigeon Company Shanghai China

**Keywords:** artificial selection, genome sweep, local environmental adaptability, pigeon, whole‐genome sequences

## Abstract

To meet human needs, domestic pigeons (*Columba livia*) with various phenotypes have been bred to provide genetic material for our research on artificial selection and local environmental adaptation. Seven pigeon breeds were resequenced and can be divided into commercial varieties (Euro‐pigeon, Shiqi, Shen King, Taishen, and Silver King), ornamental varieties (High Fliers), and local varieties (Tarim pigeon). Phylogenetic analysis based on population resequencing showed that one group contained local breeds and ornamental pigeons from China, whereas all commercial varieties were clustered together. It is revealed that the traditional Chinese ornamental pigeon is a branch of Tarim pigeon. Runs of homozygosity (ROH) and linkage disequilibrium (LD) analyses revealed significant differences in the genetic diversity of the three types of pigeons. Genome sweep analysis revealed that the selected genes of commercial breeds were related to body size, reproduction, and plumage color. The genomic imprinting genes left by the ornamental pigeon breeds were mostly related to special human facial features and muscular dystrophy. The Tarim pigeon has evolved genes related to chemical ion transport, photoreceptors, oxidative stress, organ development, and olfaction in order to adapt to local environmental stress. This research provides a molecular basis for pigeon genetic resource evaluation and genetic improvement and suggests that the understanding of adaptive evolution should integrate the effects of various natural environmental characteristics.

## 1 INTRODUCTION

Charles Darwin wrote in *The Variation of Animals and Plants under Domestication* that pigeons with diverse phenotypes are derived from the domestication and artificial selection of rock pigeons (Darwin, 1868). Modern genomic technology has verified this and provided evidence of origins in the Middle East (Shapiro et al., 2013). Archeological evidence suggests that humans exploited rock pigeons as food for >67 thousand years (Blasco et al., 2014). Human‐mediated selection can accelerate changes in animal morphological characteristics (Sendell‐Price et al., 2020), especially important economic traits (Ghoreishifar et al., 2020). Given the human need for food, showing, communication, and competition, breeders or pigeon enthusiasts have bred >350 pigeon breeds (Price, 2002). According to the classification of pigeons, breeds can be divided into four categories, including production, ornamental, competition, and experimental. The diversity of pigeon breeds offers an animal model for the study of genetics (George et al., 2020; Stringham et al., 2012), domestication (Shapiro et al., 2013), neurology (Belekhova et al., 2009; Cnotka et al., 2008; Rehk et al., 2007), and behavior (Sasaki et al., 2018). Additionally, there have been advances in understanding of the evolution, development, and genetics of intraspecific variations (Domyan & Shapiro, 2016a). Research associated with pigeon genetics generally has two main subfields: 1) analysis of the genetic mechanism of quality traits, especially the crest (Shapiro et al., 2013; Vickrey et al., 2015), foot feather (Boer et al., 2019; Domyan et al., 2016b), and plumage color (Domyan et al., 2014; Vickrey et al., 2018) and 2) analysis of quantitative traits, including determination of which homing ability in racing pigeons is a complex trait related to endurance, speed, survivability, and positioning ability, as well as navigational experience (Julia et al., 2017). Moreover, genes involved in the positively selected formation of neuromuscular junctions and the central nervous system in carrier pigeons have been identified (Caspermeyer, 2018; Gazda et al., 2018; Shao et al., 2020).

Moreover, study of adaptive genetic evolution mechanisms in relation to extreme or severe environments has increased in recent years, including the adaptability of humans and animals to high‐altitude hypoxia (Beall, 2006; X. Liu et al., 2019; Wei et al., 2016), severe cold (Fumagalli et al., 2015; Ghoreishifar et al., 2020), strong ultraviolet radiation (Bertolini et al., 2018; Flori et al., 2019; L. Yu et al., 2016), and hot and dry climates (Kim et al., 2016). *F*_ST_ & *θ_π_* ratio has been proved to be a very effective method for detecting and selecting and eliminating areas, especially when mining functional areas closely related to the living environment, it can often get a strong selection signal (Ababaikeri et al., 2020; Chen et al., 2016; Liu et al., 2020; Qiu et al., 2015). This method identified a large number of genes related to artificial selection or economic traits in other domestic animals (Li et al., 2013, 2014; Zhang et al., ,^2018^, ^2020^). *F*_ST_ is suitable for the detection of selection signals between two populations and can detect the regions where the genome is differentiated (gene frequency). The principle of *θ_π_* analysis is based on the heterozygosity, and the selected regions tend to decrease in nucleotide polymorphism. That is to say, the genomic region has both the differentiation of the genotype frequency and the reduction in nucleotide polymorphism, and it is considered that the region has been selected. These research strategies allow identification of the local environmental adaptability of pigeons and offer new insights into adaption to harsh environment and climate.

The Tarim pigeon (i.e., the Yarkant pigeon and the Kashgar pigeon) was bred over thousands of years by residents of the Yarkant River and Tarim River basins in the western part of the Tarim Basin in Xinjiang through natural breeding and domestication of feral pigeons (Liang Yayan, 2019). This breed is well‐adapted to the extreme local climate (Mainuer Slamu, 2017; Tan Rui, 2011), which is a typical warm‐temperate, continental, arid climate. The main climatic characteristics are dry heat, severe cold, and strong solar radiation, with large daily and annual temperature ranges (Abdul Rexiti Aji, 2002). Because of the pursuit of economic benefits, modern large‐scale pigeon breeding enterprises mostly choose commercial pigeon varieties for production, such as King pigeons (Ye et al., 2018) and Euro‐pigeons (Pomianowski et al., 2009), which have the advantages of a white carcass, large size, and high fecundity.

Here, we generated the whole‐genome sequences of 52 pigeons from seven breeds, including one local breed, one high‐flying breed, and five commercial meat‐types, by selective sweep and performing combined calculations for the *F*_ST_ and log_2_(*θ_π_* ratio) values. We observed strong selection markers near genes known to be artificially selected for plumage color, growth, and development in other animals. Importantly, we discovered genetic pathways related to local environmental adaptability in the Tarim pigeon.

## 2 MATERIALS AND METHODS

### 2.1 Pigeons

**FIGURE 1:**
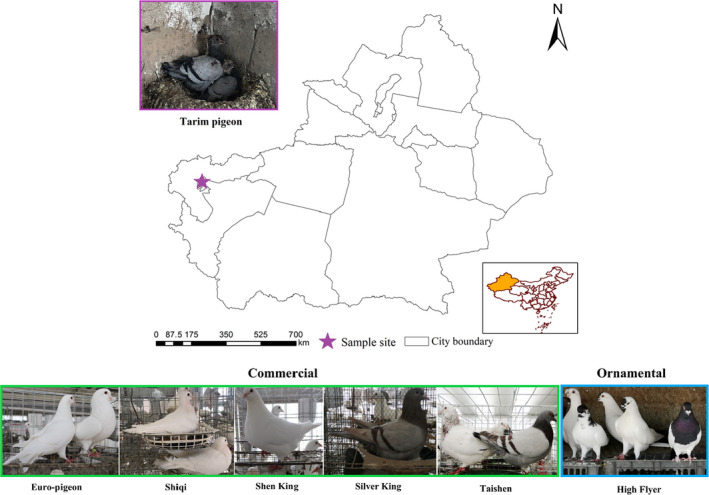
Phenotype of the seven pigeon breeds and geographical origins of local breeds

The five commercial meat breeds included seven Euro‐pigeons (EU; Shanghai Jinhuang Pigeon Industry Co., Ltd.), eight Silver Kings, seven Shiqi, eight Shen Kings, and eight Taishen self‐distinguishing male and female pigeons (WK, SQ, SK, and TS; Shenzhen Tianxiang Da Pigeon Co., Ltd). Six high fliers (HF) were from Shanghai Jinhuang Pigeon Industry Co., Ltd., and eight Tarim pigeons (TR) were from Mushi Township, Shufu County, Kashgar, Xinjiang (Figure 1). Pigeons used in this study were approved by the Ethics and Animal Welfare Committee of Shanghai Academy of Agricultural Sciences (No. SAASPZ0521012). For each pigeon, genomic DNA was extracted from blood samples using a Tiangen DNA extraction kit, and sequencing was performed on an Illumina HiSeq PE150 systems (Illumina, Carlsbad, CA, USA).

### 2.2 Alignment against the reference genome

High‐quality sequencing data were aligned with the reference genome (https://www.ncbi.nlm.nih.gov/genome/10719?genome_assembly_id=39619) using BWA software (parameter: mem‐t4‐k32‐m) (Li & Durbin, 2009), and the results were analyzed using SAMTOOLS (parameter: rmdup) (Li et al., 2009).

### 2.3 SNP detection and annotation

We used SAMtools to detect population SNPs (Li et al., 2009) and a Bayesian model to detect polymorphic sites in the population. High‐quality SNPs were obtained by filtering and screening as follows:

1. Q20 quality control (filter SNPs with quality values with a sequencing error rate >1%).
2. SNP sites separated by at least 5 bp.
3. SNP coverage depth ranging from 1‐, 3‐, or 5‐fold the average depth.

ANNOVAR software was used to annotate the SNP results (Wang et al., 2010).

### 2.4 Analysis of genetic background in the population

The distance matrix was calculated using TreeBeST software (v.1.9.2; http://treesoft.sourceforge.net/treebest.shtml), and phylogenetic trees were constructed using the neighbor‐joining (NJ) method. The bootstrap values were obtained after 1,000 calculations. We used EIGENSOFT (v5.0; https://www.hsph.harvard.edu/alkes‐price/software/) for principal component analysis on an individual scale for the 52 pigeons (Patterson et al., 2006). Use PLINK to analyze population structure, create PLINK input file—Ped file, and then use FRAPPE v1.1 to estimate individual admixture (Tang et al., 2005).

### 2.5 Analyses of regions of homozygosity (ROHs) and linkage disequilibrium (LD)

Regions of homozygosity for each pigeon breed were identified using PLINK (v.1.07) (Purcell et al., 2007). The parameters of ROH analysis are as follows (plink ‐‐ file result ‐‐ allow extra chr ‐‐ homozyg SNP 100 ‐‐ homozyg kb 500 ‐‐ homozyg density 50 ‐‐ homozyg gap 1000 ‐‐ homozyg window SNP 50 ‐‐ homozyg window het 2 ‐‐ homozyg window missing 5 ‐‐ out roh; rm *. Nosex). Based on the detected ROHs, the genomic inbreeding coefficient (*F_ROH_*) of pigeons was calculated (McQuillan et al., 2008):

$$F_{ROH}=\frac{L_{ROH}}{L_{AUTO}}$$

Among them, *L*_ROH_ represents the total length of ROH fragment on autosomal, and *L*_AUTO_ is the total length of autosome.

To evaluate LD decay, the squared correlation (*r* ^2^) between any two loci was calculated using Haploview (v.4.2) (Barrett et al., 2005). LD analysis parameters are as follows (‐n –dprime‐minMAF 0.05). The average r^2^ value was calculated for pairwise markers in a 500‐kb window and averaged across the whole genome.

### 2.6 Genome‐wide selective‐sweep test

*F*_ST_ and *θ_π_* have proven effective for detecting selective elimination regions, especially when mining functional regions closely related to the living environment, when strong selection signals can be obtained. We calculated the genome‐wide distribution of *F*_ST_ values (Weir & Cockerham, 1984) and *θ_π_* ratios among the seven pigeon breeds, using a sliding‐window approach (40‐kb windows with 20‐kb increments). The *θ_π_* ratios were log_2_(*θ_π_* ratio) transformed. We considered the windows with the top 5% values for the *F*_ST_ and log_2_(*θ_π_* ratio) simultaneously as candidate outliers under strong selective sweeps (Li et al., 2013) (Table S1). *F*_ST_ and log_2_(*θ_π_* ratio) joint analysis including TR. VS. EU, TR. VS. SQ, and TR. VS. SK revealed the genomic region imprint of three commercial white feather pigeon breeds. TR. VS. TS was used to analyze the selection of TS, and TR. VS. WK was used to reveal the selection of WK. The other six varieties were used as controls to detect the selection signal of HF. Five commercial meat pigeon breeds were used as control to study the adaptive selection of Tarim pigeon. Furthermore, the overlap information of a selected signal was obtained using the “vennDiagram” package in R (https://www.omicstudio.cn/tool/6).

### 2.7 Candidate gene analysis

We compared *F*_ST_ and log_2_(*θ_π_* ratio) values of the selective genomic regions with those at the whole‐genome scale for TR pigeons. Overlaps of candidate genes were identified in extreme environments. Gene Ontology (GO) analysis was performed using R packages (GOseq and topGO), and Kyoto Encyclopedia of Genes and Genomes (KEGG) analysis was performed using Kobas software (http://kobas.cbi.pku.edu.cn/kobas3/).

## 3 RESULTS

### 3.1 Genome sequencing and mapping

After filtering the sequencing data, high‐quality clean data were obtained. The data of 52 samples were statistically analyzed (Table S2), including sequencing data output, sequencing error rate, Q20 content (%), Q30 content (%), and GC content (%). The total amount of sequencing data is 491.6 Gb, and the clean data are 490.7 Gb. The genome size is 1107989085 bp, the average mapping rate of population samples is 97.5%, the average sequencing depth of genome is 7.85, and the average coverage 1X is 98.4% (Table S3). Finally, a total of 5,673,290 SNPs with high quality were obtained (Table S4), and 26,640 genes were annotated (Table S5).

### 3.2 Population genetics

**FIGURE 2:**
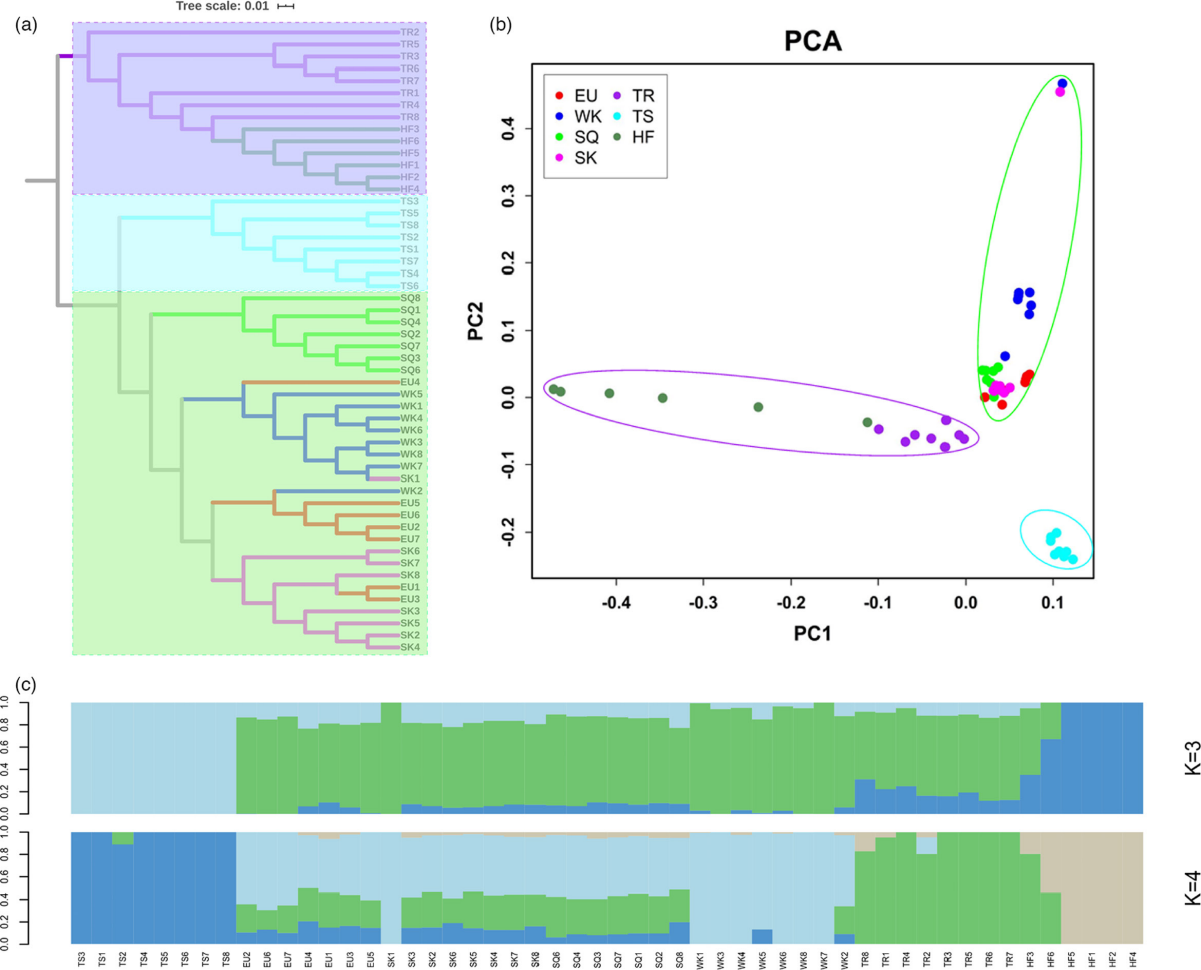
(a) NJ phylogenetic tree for the seven pigeon breeds. European pigeon (EU); Silver King (WK); Shiqi (SQ); Shen King (SK); Tarim pigeon (TR); Taishen (TS); High Flier (HF). (b) Two‐ dimensional PCA plot of pigeon breeds. (c) Genetic structure of pigeon breeds according to ADMIXTURE, with K = 3, 4

Neighbor‐joining tree analysis supported two of the separate clusters, including Chinese breeds and commercial breeds (Figure 2a). PCA results revealed strong clustering of seven pigeon breeds into three genetic groups, with HF and TR clustered in a group, EU, SK, WK, and SQ clustered together, and TS separated into a single group (Figure 2b). ADMIXTURE analysis confirmed that three ancestral groups were formed (Figure 2c).

**FIGURE 3:**
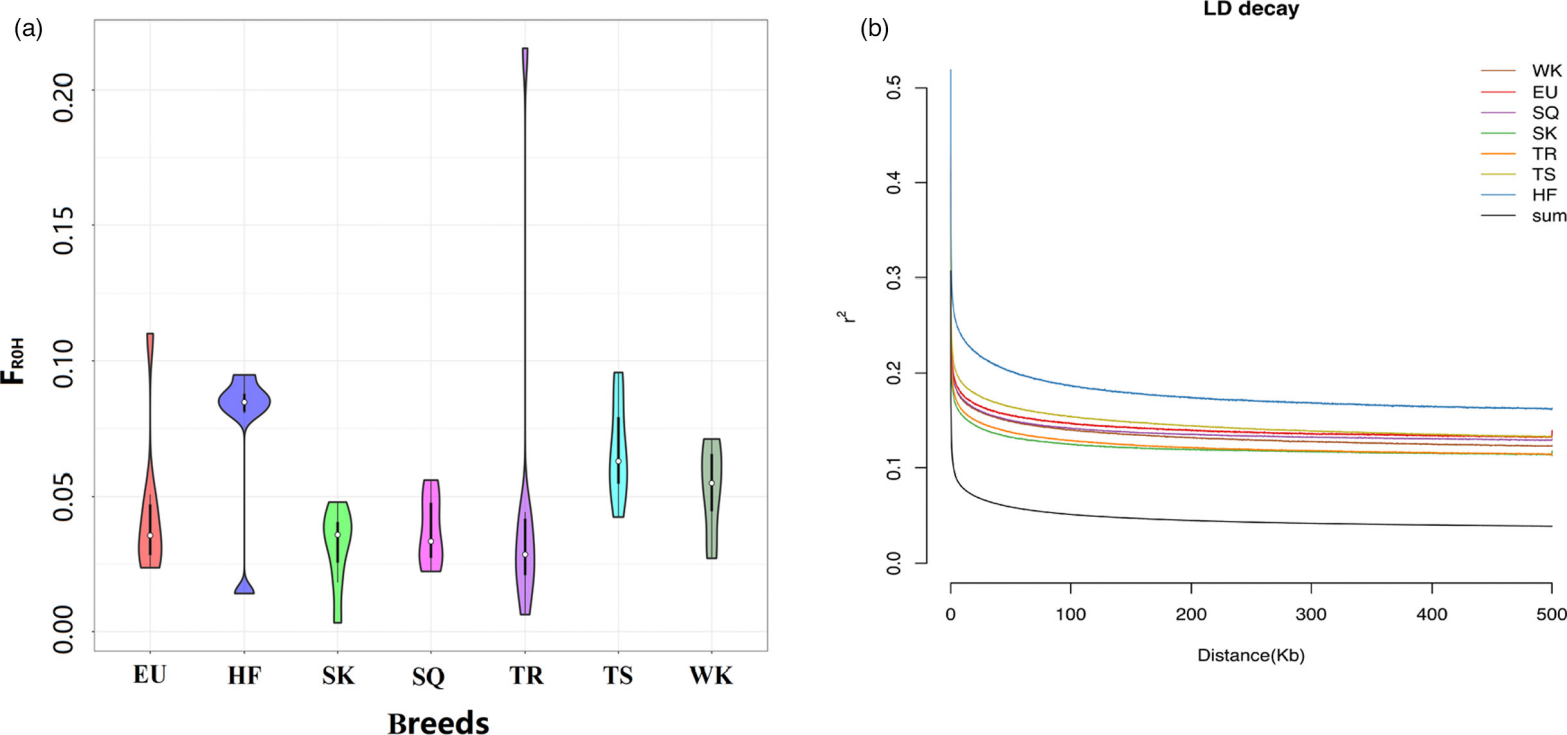
(a) Analysis of the *F*_ROH_ of the seven pigeon breeds. (b) LD decay of the seven pigeon populations, as measured by *r* ^2^

The genomic variations [average ROHs, mean ROH size, and the inbreeding coefficient (*F*_ROH_)] for the seven pigeon groups showed congruent patterns. Chinese HF breeds showed an extreme inbreeding coefficient (*F*_ROH_) and exhibited the most ROHs (96) and the largest ROH size (76,829.6 kb), whereas the greatest genomic variations were observed in the TR breed from Kashgar in Xinjiang (Figure 3a, Table S6). The TR and SK pigeon breeds showed an overall high level of genetic diversity and rapid LD decay, whereas the HF breeds, as a Chinese traditional pet pigeon, exhibited lower genetic diversity and slower LD decay (Figure 3b).

### 3.3 Selective‐sweep signals in commercial pigeon breeds

#### 3.3.1 Plumage color

**FIGURE 4:**
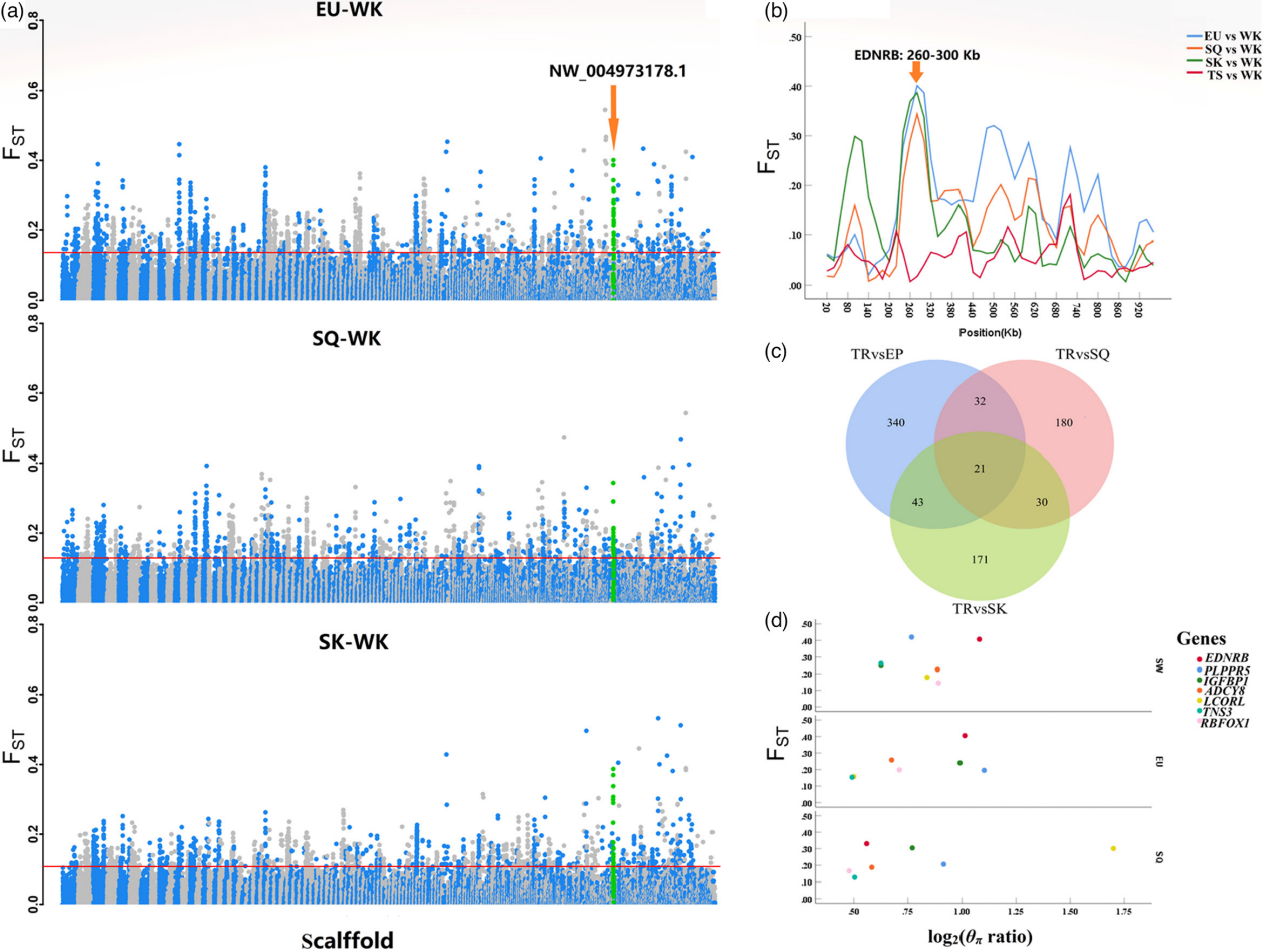
(a) *F*_ST_ plot of the comparison of the white pigeon with WK. The threshold line represents the top 1% of the *F*_ST_ value. (b) *F*_ST_ corresponding to the selective sweep of white plumage color on scaffold NW_004973178.1 encompassing *EDNRB*. (c) Venn diagram showing the shared gene number between three white commercial pigeon breeds, with local TR pigeons used as the control group (*F*_ST_ and *θ_π_* analyses). (d) Seven key genes were selected as associated with white plum

Using WK as the control group, 303, 181, and 190 gene regions were identified by *F*_ST_ and log_2_(*θ_π_* ratio) analyses in EU, SQ, and SK, respectively (Table S7). Furthermore, nine genes were identified by overlap analysis, among which *EDNRB* was associated with white plumage color (Figure 4a and b, Table S8). With white pigeons and TR pigeons as the control group, a total of 118 overlaps were identified in TS pigeons (Table S9), among which genes enriched for the KEGG pathway melanogenesis (ID: clv04916) included *AHCY*, *ASIP*, *WNT6*, and *WNT10A*. When compared with the HF pigeons, *ASIP* was identified as related to feather color with WK. Other genes related to WK plumage color included *CREB1* and *SLC45A1*.

#### 3.3.2 Production

Compared with the TR pigeon, 21 genes in the white‐feathered meat pigeon were identified as related to body size according to overlap analysis, including 7 definite named genes (*EDNRB*, *PLPPR5*, *RBFOX1*, *IGFBP1*, *TNS3*, *ADCY8*, and *LCORL*) (Figure 4c and d, Table S10). Additionally, *LCORL* was identified in TS pigeons, whereas *TNS3* and *IGFBP1* genes were identified in WK pigeons. We speculated that these three genes are related to body size traits of commercial pigeon breeds. Selective signal analysis revealed that *RAMP3*, *KIAA0556*, *VAMP3*, and *GSG1L* play key roles in WK reproduction. Compared with the TR pigeons of all commercial meat pigeon breeds, overlap analysis identified four genes (Table S11), with *RBFOX1* shared between breeds and related to human neuronal development (Fogel et al., 2012).

### 3.4 Selective‐sweep signals in High Fliers

The HF are a highly cultivated breed with a breeding history of >300 years in China. Selection signals and overlap analysis revealed that 73 gene regions received strong artificial selection or showed the lowest genetic diversity (Table S12), with all of these genes annotated in the human phenotype ontology database (http://www.webgestalt.org/). HPO enrichment analysis revealed their association with facial features and muscular dystrophy (Tables 1, S13). Genes associated with pigment deposition included *MC1R*.

**TABLE 1:** HPO annotation of candidate genes

Term ID	Term name	Intersections
HP:0000152	Abnormality of head or neck	*SYNE1*, *DMD*, *SOS1*, *CTDP1*, *TLR3*, *BRAF*, *GRIA4*, *SGCD*, *COG8*, *CDH1*, *GAS8*, *TUBB3*
HP:0002817	Abnormality of the upper limb	*SYNE1*, *DMD*, *SOS1*, *CTDP1*, *BRAF*, *SGCD*, *COG8*, *GAS8*, *TUBB3*
HP:0011805	Abnormal skeletal muscle morphology	*SYNE1*, *DMD*, *SOS1*, *CTDP1*, *BRAF*, *GRIA4*, *SGCD*, *COG8*, *TUBB3*
HP:0000271	Abnormality of the face	*SYNE1*, *DMD*, *SOS1*, *CTDP1*, *BRAF*, *SGCD*, *COG8*, *CDH1*, *GAS8*, *TUBB3*
HP:0011821	Abnormality of facial skeleton	*SOS1*, *CTDP1*, *BRAF*, *COG8*, *CDH1*, *GAS8*, *TUBB3*
HP:0000492	Abnormal eyelid morphology	*DMD*, *SOS1*, *CTDP1*, *BRAF*, *COG8*, *CDH1*, *TUBB3*
HP:0030319	Weakness of facial musculature	*SYNE1*, *DMD*, *SGCD*, *TUBB3*
HP:0003560	Muscular dystrophy	*SYNE1*, *DMD*, *SGCD*
HP:0002212	Curly hair	*SOS1*, *BRAF*

### 3.5 Selective‐sweep signals in Tarim pigeons

**FIGURE 5:**
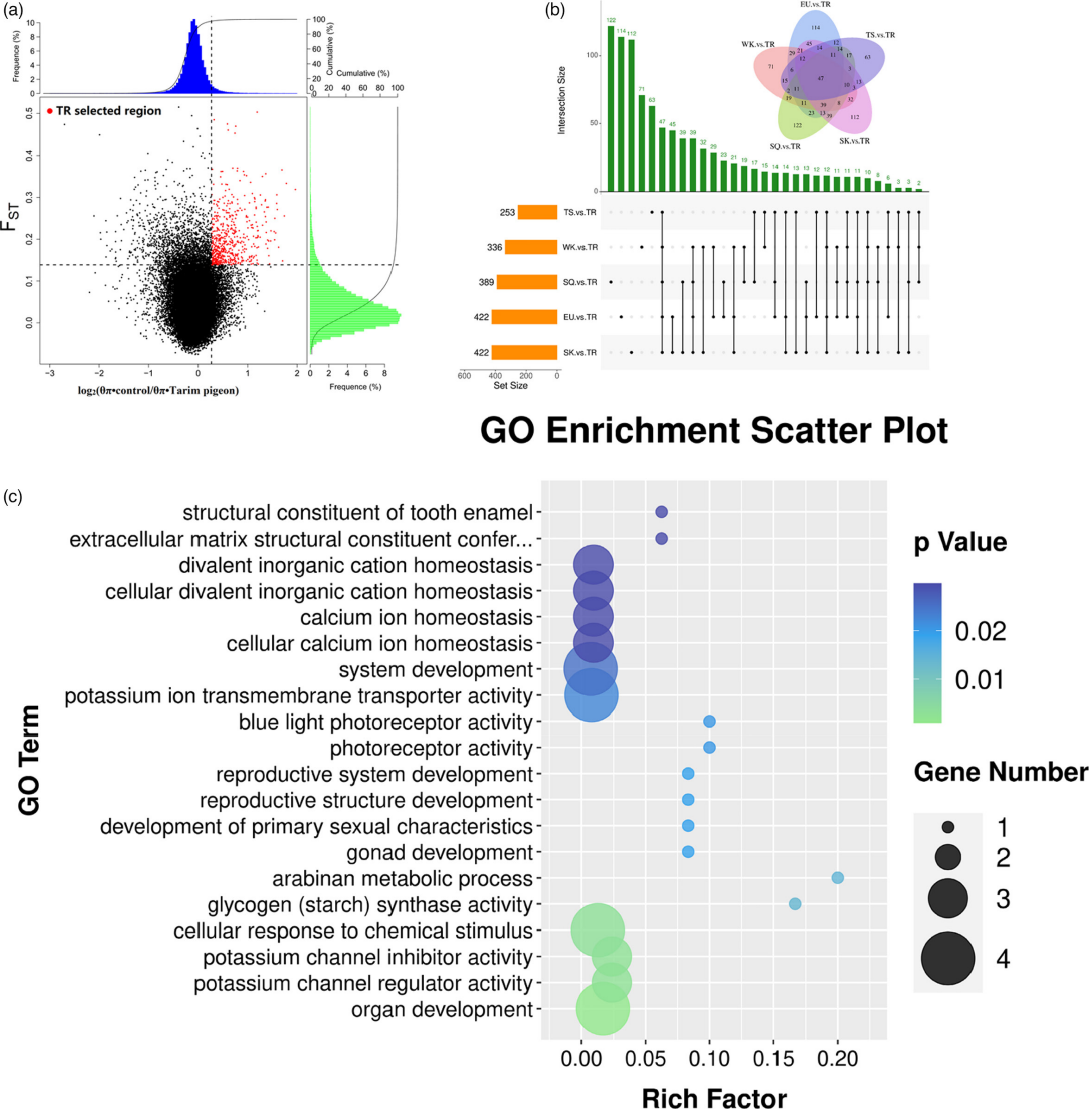
(a) The top 5% of distribution of log_2_ values (*θ_π_* •control/*θ_π_* •Tarim pigeon) and highest *F*_ST_ values calculated in 40‐kb sliding windows with 20‐kb increments between the Tarim pigeon and the control commercial population. (b) Upset and Venn diagrams show the overlapping genes between TR and the five commercial breeds. (c) Enriched GO terms for key genes detected by selective sweep according to the *F*_ST_ and *θ_π_* ratio

Rapidly evolving adaptive genes related to environmental stress were used as the windows to identify the top 5% (*F*_ST_ and the log_2_(*θ_π_* ratio)) of genes as the candidate outliers of strong selective sweeps (Figure 5a). Compared with the five commercial varieties, positive selection genes were identified in TR, including 422, 389, 422, 253, and 336 genes related to adaptability (Table S14). Additionally, 47 gene regions were identified by overlap analysis (Figure 5b, Table S15), and GO analysis revealed significant enrichment of 22 candidate genes embedded in the selective regions (169 GO terms; *p *< 0.05) (Tables S16, S17), including organ development (GO:0048513, *p *= 0.001), potassium channel inhibitor activity (GO:0019870, *p *= 0.003), cellular response to chemical stimulus (GO:0070887, *p *= 0.003), reproductive structure development (GO:0048608, *p *= 0.02), and blue light photoreceptor activity (GO:0009882, *p *= 0.02) (Figure 5c). These enriched genes were related to adaptability, including strong solar radiation, large diurnal range, and alkaline water. According to the selected regions, the enrichment pathways were mainly related to abiotic factors (physical and chemical environments), as well as organ development and olfactory and visual systems. The KEGG pathways enriched by the selected genes of TR include Adipocytokine Signaling Pathway (ID: clv04920, *p *= 0.03) and Insulin Signaling Pathway (ID: clv04910) (Table S18, Figure S1). Both pathways involve *PPARGC1A* gene, which plays a key role in adaptive thermogenesis.

## 4 DISCUSSION

The genetic architecture plays a fundamental role in the origin and maintenance of local adaptation with gene flow (Tigano & Friesen, 2016). Here, phylogenetic tree mainly includes two branches: one for commercial pigeons and mostly introduced and cultivated varieties; and the other comprises TR and HF pigeons, indicating that traditional Chinese ornamental pigeons might originate from local breeds. The results of ROH analysis of the high‐flying pigeon breed revealed their being highly inbred. The artificial high‐intensity and continuous selection requires that this group maintain specific morphological characteristics for breeding, such as a short beak and lightweight. Meat pigeon breeds can provide humans with high‐quality protein sources, mainly including meat and egg products. Artificial selection targets mainly focus on body size and fertility, and large‐scale cages are often used. These pigeon genetic resources provided a more extensive phenotypic basis for research purposes. The results are mainly classified into two categories: economic traits and local environmental adaptability. Economic traits mainly include feather color, body size, fecundity, and ornamental traits, whereas environmental adaptability abiotic factors include physical environment (light, temperature, etc.) and chemical environment (water, food, air, etc.).

### 4.1 Economic traits

#### 4.1.1 Genes related to plumage color

Compared with colored feather pigeons, white feather pigeons have better carcass appearance after slaughtering, so they occupy the mainstream market in the world. Comparative analysis between white‐feathered pigeons and other colored pigeons revealed that the *EDNRB* gene region showed a strong selection signal. This gene is widely reported as related to mammalian skin, coat, and iris pigmentation, including several human syndromes with eye‐color defects and heterochromia (Issa et al., 2017; Morimoto et al., 2018), iris pigmentation, heterochromia patterns, and coat color in pigs (Moscatelli et al., 2020; Wilkinson et al., 2013). Additionally, a missense mutation in *EDNRB* causes lethal white foal syndrome in horses (Metallinos et al., 1998; Santschi et al., 1998). In birds, only sporadic reports of this gene variation have been associated with feather color of quails (Miwa et al., 2006, 2007). Pigeons with white plumage usually have dark brown iris color. The results of selective signal scanning suggested that *EDNRB* might play a key role in pigeon feather and iris color.

As a monotypic bird, the sex identification of pigeons is difficult in the commercial production of meat pigeons. TS pigeons are one of the a few breeds in which males and females can be identified by feather color. For almond‐colored pigeons with sex‐linked, copy number variation analysis identified a genomic region that include *MLANA* (Bruders et al., 2020). Moreover, homozygous almond males (ZSt/ZSt) develop severe eye defects and often lack plumage pigmentation, suggesting that higher dosage of the mutant allele is deleterious (Bruders et al., 2020). In this study, the genomic region controlling pigment deposition in TS, which includes *AHCY*, *ASIP*, *WNT6*, and *WNT10A*. Multiple studies report the involvement of *ASIP* in determining coat color in domestic animals, including sheep (Zhang, Li, et al., 2017), horse (Grilz‐Seger et al., 2019), donkey (Abitbol et al., 2015), cattle (Xu et al., 2015), and buffalo (Liang et al., 2020). Additionally, variation in the 5′ region of the gene in Japanese quails can cause feather color dilution (Robic et al., 2019), and the expression of the gene is associated with chicken sexual dimorphism (Oribe et al., 2012). Moreover, *WNT6* and *WNT10A* are related to the color pattern of butterfly wings (Martin & Reed, 2014), and subsequent studies showed that *WNT6* is involved in sex‐related feather color patterns (Iijima et al., 2019). Furthermore, *WNT10A* is associated with hypohidrotic ectodermal dysplasia, which is usually inherited in an X‐linked form and features light pigmentation (Wright et al., 1993).

In the WK pigeon population, *ASIP* was only identified in high‐flying pigeons, whereas *SLC45A1* and *CREB1* were identified in comparisons with other groups. *SLC45A1* encodes a protein that plays a role in glucose uptake and is implicated in the regulation of glucose homoeostasis in the brain (Bartölke et al., 2014). There are few reports on the relationship between *SLC45A1* and pigment deposition, although *SLC45A2* is associated with skin and hair pigmentation (Fracasso et al., 2016), and its mutation is related to chicken Silver Feather (Gunnarsson et al., 2007). Additionally, *SLC45A2* mutation is associated with classical color phenotypes of pigeons and offers a mechanistic explanation of their dominance and epistatic relationships (Domyan et al., 2014). Furthermore, evidence indicates that inherited abnormalities in *CREB1* pigmentation‐related genes can not only increase the risk of cutaneous melanoma but also influence patient clinicopathological features (Loureno et al., 2020).

We identified *MC1R* in HF during comparison with other breeds and encodes a protein that regulates pigment deposition (Mountjoy et al., 1992). Variation in *MC1R* in different species can lead to a dominant black‐coat color phenotype or recessive red or yellow phenotype (Robbins et al., 1993). In pigeons, variation in *MC1R* resulting in a V85 M substitution is associated with phenomenalism (Guernsey et al., 2013).

#### 4.1.2 Genes related to production

The production traits of meat pigeons mainly include meat traits and reproductive traits. Commercial pigeon breeders pursue larger body size and higher meat production, mainly based on the sales of squab at 28 days old. The weight of commercial meat pigeons is generally 500–800 g, while that of Chinese traditional ornamental pigeons and local pigeons is only about 350 g. Compared with local breeds, we identified *LCORL*, *TNS3*, and *IGFBP1* as associated with growth and development in white meat pigeons. The *LCORL* region has been identified as a locus for human height (Wood et al., 2014) and weight (Horikoshi et al., 2013), and genome‐wide analysis of multiple species revealed this region as related to body size, including in pig (Rubin et al., 2012), horse (Srikanth et al., 2019), cattle (Xu et al., 2015), and sheep (Yurchenko et al., 2019). The present study is the first identification of this gene as being related to production in pigeons. *TNS3* plays an important role in development and growth, and its inactivation in mice results in growth retardation and postnatal lethality (Chiang et al., 2005). *TNS3* gene was enriched in muscle organ development (GO: 0007517) and muscle structure development (GO: 0061061), and *TNS3* is associated with fossil bone length in Pekin ducks (Deng et al., 2019). *IGFBP1* is secreted by hepatocytes and other cell types (Baxter, 2014), and studies report that women with elevated *IGFBP1* levels are more likely to have a low relative muscle mass (Stilling et al., 2017).

Compared with other breeds, we identified reproduction‐related genes in WK, including *RAMP3*, *KIAA0556*, *VAMP3*, and *GSG1L*. *RAMP3* encodes an adrenomedullin receptor (McLatchie et al., 1998) involved in uterine quiescence during pregnancy and regulated by steroid hormones (Thota et al., 2003). Additionally, *RAMP3* expression correlates with temporary sperm storage in and possible sequential sperm release from the chicken uterovaginal junction following artificial insemination (Yang et al., 2020). Balanced chromosome rearrangements in unaffected individuals possess high reproductive risks, including infertility, abnormal offspring, and pregnancy loss, with *KIAA0556* detected in families harboring these rearrangements (Tan et al., 2020). *GSG1L* and *VAMP3* play important roles in the development of animal gonads (Boonanuntanasarn et al., 2020), and *VAMP3* is important to sperm fertilization in pig (Tsai et al., 2010) and associated with testis weight in chickens (Zhang, Yu, et al., 2017).

#### 4.1.3 Genes related to ornamental traits

Compared with other breeds, the genes identified in high‐flying pigeons are mostly related to rare human diseases, especially muscular dystrophy and special facial features. This might be related to the direction chosen by breeders, such as a light body, short beak, big eyes, and feather color. *SYNE1* is associated with Emery–Dreifuss muscular dystrophy (Chen et al., 2017; Heller et al., 2020; Sandra et al., 2019), and mutation of *DMD*, as the largest in the human genome (total intron content: >2.2 Mb) (Keegan, 2020), causes Duchenne muscular dystrophy and Becker muscular dystrophy (Yang et al., 2019). *SOS1* and *BRAF* are associated with Noonan syndrome, an autosomal‐dominant disorder characterized by short stature, congenital heart disease, curly hair, and facial dysmorphia (Allanson & Roberts, 2001; El Bouchikhi et al., 2016; Quaio et al., 2013). *CTDP1* is the only gene in which pathogenic variants are known to cause congenital cataracts, facial dysmorphism, and neuropathy characterized by abnormalities of the eye (Kalaydjieva & Chamova, 1993). *SGCD* is associated with recessive limb‐girdle muscular weakness and Pompeii disease (Bevilacqua et al., 2020).

### 4.2 Adaptive traits

Compared with commercial pigeon breeds and in addition to selection pressures for meat and fertility, local TR pigeons had to adapt to a warm arid climate in Kashgar (Liang et al., 2019) characterized by strong solar radiation, and large daily and annual temperature ranges (Abdul Rexiti Aji, 2002). Additionally, sulfate concentrations exceed the groundwater quality standard in 73.2% of the unconfined groundwater area and in 53.2% of the confined groundwater area in Kashgar Delta (Wei et al., 2019). Studies have focused on plant soil chemical tolerance (Bian et al., 2020), and a recent report demonstrated the adaptability of limestone langurs to a calcium ion chemical environment (Liu et al., 2020). *KLHL8* was enriched in a pathway related to sulfate transmembrane transporter activity (GO:0015116, *p *= 0.03). *GLRA2* is a member of the ligand‐gated ion channel superfamily (Feng et al., 2001) that plays an important role in maintaining potassium and calcium homeostasis. It is speculated that these genes may be related to the water environmental adaptability of TR pigeon.

*PPARGC1A* (also known as *PGC*‐*1α*) is related to metabolism and starch synthesis by encoding a nuclear transcriptional coactivator that plays a pivotal role in metabolic processes, including mitochondrial biogenesis, thermogenesis, respiration, insulin, secretion, and gluconeogenesis (Baar, 2014). Human variants of *PPARGC1A* are associated with endurance (Ahmetov et al., 2016) and might be involved in pigeon energy maintenance. Moreover, *PPARGC1A*, *METTL15*, and *PGAM5* play an important role in mitochondria (Magalhaes et al., 2018; Van Haute et al., 2019; Yu et al., 2020), with deletion of *PGAM5* resulting in accelerated retinal pigment epithelial senescence *in vitro* and *in vivo* (Greenway et al., 2020). Furthermore, *ADGRL3* expression is highly regulated during brain development and peaks during the prenatal and postnatal periods (Arcos‐Burgos et al., 2010), with numerous studies reporting that *ADGRL3 (LPHN3)* is related to attention‐deficit/hyperactivity disorder (Acosta et al., 2016; Huang et al., 2019; Hwang et al., 2015). *ADGRL3* gene is enriched in the blue light photoreceptor activity (GO:0009882, *p *= 0.02). Photoreceptors are essential for circadian rhythm in animals. It is speculated that this gene may be related to the rhythmic behavior of TR pigeons. *NTNG1* is associated with neurological diseases, including Rett syndrome (Archer et al., 2006), schizophrenia, and bipolar disorder (Eastwood & Harrison, 2008; Wilcox & Quadri, 2014). In the present study, we found that pathways enriched in the selected gene regions of TR pigeons were mainly concentrated in organ development, inorganic ion transport, circadian rhythm, the olfactory system, and metabolism.

## 5 CONCLUSIONS

This study elucidated the genetic and evolutionary relationship among seven pigeon breeds. Inbreeding or genetic diversity assessment revealed the degree of artificial selection of ornamental breeds, local breeds, and commercial pigeon breeds. The genomic imprints left by artificial selection revealed the *EDNRB* gene that affects white feather traits and the *LCORL* gene that affects body weight or size, which can be used as candidate genes for molecular breeding of meat pigeons. Compared with the choices of pigeons in production and ornamental traits, the adaptability of human‐mediated selection was more complex and diverse, including chemical and physical environments. This research insights into how the Tarim pigeon has evolved an effective adaptive mechanism to cope with the adverse conditions of local drought, brackish water, strong light, and large temperature differences. Screening genomic regions of pigeons related to economic traits and adaptability not only broadens the understanding of selection and evolutionary processes in pigeons but also helps make better use of genetic resources for breeding improvements.

## CONFLICT OF INTEREST

The authors declare that they have no conflict of interest.

## Supporting information

Figure S1

Table S1

Table S2

Table S3

Table S4

Table S5

Table S6

Table S7

Table S8

Table S9

Table S10

Table S11

Table S12

Table S13

Table S14

Table S15

Table S16

Table S17

Table S18

## Data Availability

The data that support the findings of this study are available from the corresponding author upon reasonable request.
