# Supplementary figures and images for: Whole‐genome sequencing reveals the artificial selection and local environmental adaptability of pigeons (Columba livia)
Source: Evol Appl. 2021 Aug 5;15(4):603–17. doi: 10.1111/eva.13284 (PMC9046921; doi:10.1111/eva.13284)

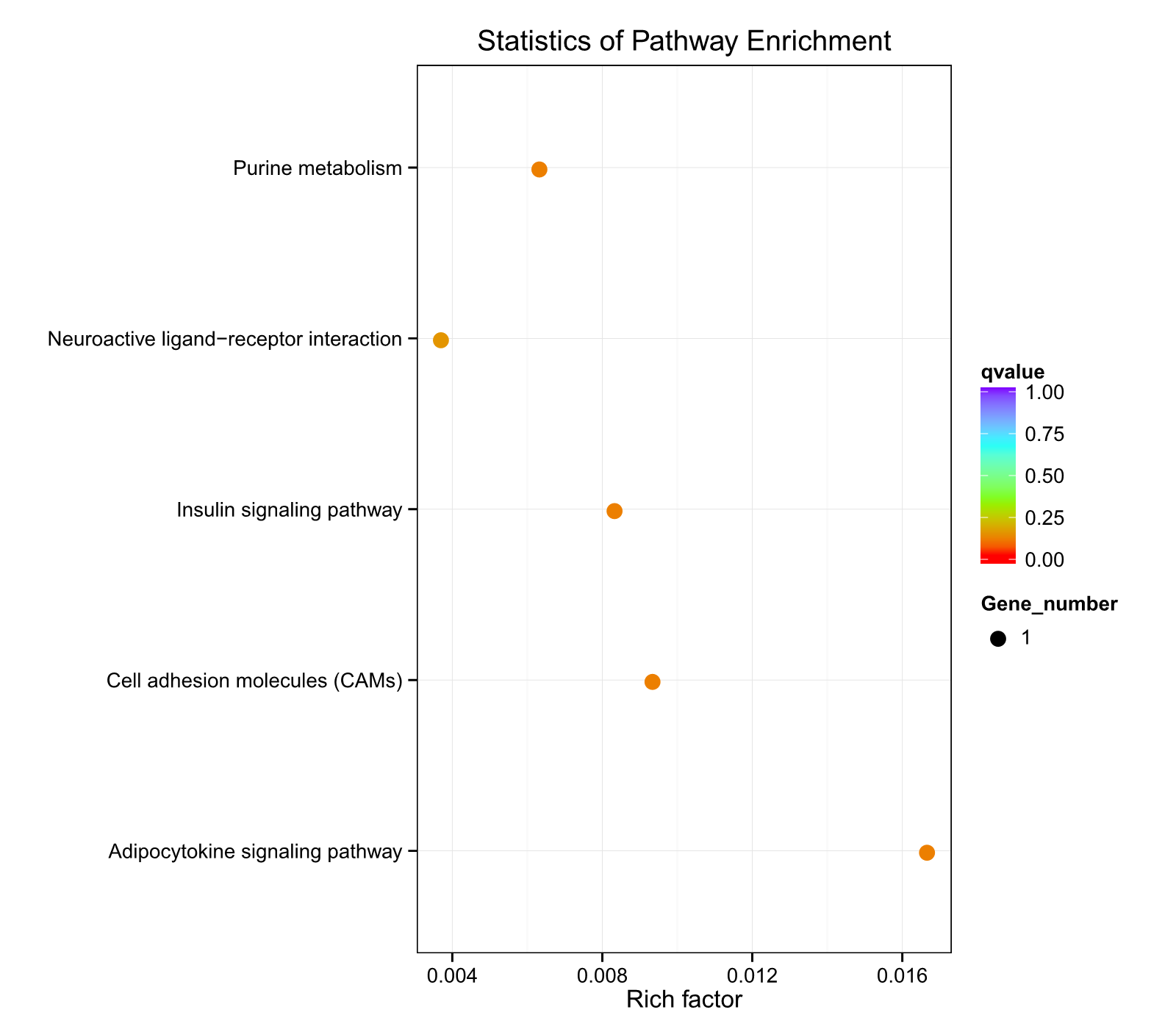

Supplement: Supplementary file 1 — Figure S1 [file EVA-15-603-s007.png]
